# Assessment of Psychological Consequences of Violence in Psychiatric Nurses

**DOI:** 10.7759/cureus.48025

**Published:** 2023-10-31

**Authors:** Mamdouh Alamri, Waleed Almalki, Naif T Almagthly, Khalil M Al-Harbi, Mohammad H Almutairi

**Affiliations:** 1 Psychiatry, Ministry of Health, Riyadh, SAU; 2 Nursing, Erada and Mental Health Complex, Jeddah, SAU; 3 Addiction Treatment and Mental Health, Erada and Mental Health Complex, Jeddah, SAU; 4 Medical Services Administration, University of Jeddah, Jeddah, SAU; 5 Psychiatric Mental Health, Erada and Mental Health Complex, Jeddah, SAU

**Keywords:** kingdom of saudi arabia (ksa), psychological consequences, psychiatric nurses, violence prevention, violence

## Abstract

Background: Workplace violence, particularly within psychiatric nursing environments, is an emerging concern globally and has been recognized as a significant occupational stressor with considerable psychological consequences, yet it remains underexplored, warranting an in-depth study.

Aim: This study aimed to assess the psychological consequences of violence on psychiatric nurses in Jeddah, Saudi Arabia.

Method: A descriptive, cross-sectional design was used. A convenience sampling technique was used to select 198 psychiatric nurses working in two hospitals with psychiatric departments in Jeddah. A self-administered questionnaire and the Impact of Event Scale - Revised (IES-R) were used for data collection. Data were analyzed using SPSS Statistics version 22 (IBM Corp. Released 2013. IBM SPSS Statistics for Windows, Version 22.0. Armonk, NY: IBM Corp.).

Results: Out of the 198 psychiatric nurses who participated in the study, 80.8% reported experiencing violence in their workplace, highlighting the need for effective interventions to prevent and manage violence in psychiatric settings. The majority of the participants had a diploma (68.2%), and 54.5% of the nurses had more than 10 years of experience. Among patient-related factors related to violence, drug abuse had the highest mean score of 4.07 (SD=0.89) and was ranked as the most significant factor, while among nursing-related factors, a lower level of experience in psychiatric care had the lowest mean score of 3.43 (SD=1.11) and was ranked as the least important factor. The nurses tended to avoid discussing the traumatic event, as indicated by the highest mean score of 2.10 (SD=1.18) for item 22, "I tried not to talk about it." The mean score for the avoidance subscale of the IES-R was 1.55 (SD=0.78), the highest mean score among the subscales and ranked first in terms of severity.

Conclusion: This study highlighted the significant psychological consequences of violence on psychiatric nurses in Jeddah, Saudi Arabia.

## Introduction

WHO characterizes violence as the application of physical power, either actual or threatened, toward oneself or others, which may lead to harm, trauma, fatality, or deprivation. Workplace aggression, as specified, consists of belligerent and abusive actions targeted at personnel, coupled with menacing activities in the occupational context that pose explicit or tacit threats to their professional safety, overall wellness, and health [1]. Workplace violence is defined by the National Institute for Occupational Safety and Health as “physical violence, violent behaviors and attitudes, psychological abuse (e.g., intimidation and harassment), stalking, and psychological trauma in the workplace” [2]. According to Al-Omari et al., violence is classified into three types based on who perpetrated it: self-directed, interpersonal, and collective. It is further classified into five types based on the nature of the violence, namely, physical, sexual, psychological, deprivation, and neglect [3]. Workplace violence varies from the use of derogatory words to deaths, including beatings, weeping, suicides, psychological traumas, threats over the phone, and numerous forms of harassment [3]. The Occupational Safety and Health Administration reveals that annually, about 2 million psychiatric nurses become targets of aggression at their workplaces [4]. Workplace violence is a pervasive issue, with its incidence speculated to span from 58% among nursing personnel in Anglo territories to 38% among their European counterparts [4]. The incidence of violence, particularly when preventative measures are ineffective, indicates an uncomfortable environment and a risk to workers' healthcare [1]. Workplace aggression can have profound physical implications for psychiatric nurses, manifesting both immediate and sustained effects [5]. Additionally, there is an elevated likelihood for women subjected to workplace abuse to experience mental health disorders [6]. In Saudi Arabia, numerous investigations have been conducted concerning workplace aggression among nurses [7-10]. Psychiatric nurses are often exposed to both physical and psychological violence by patients in healthcare systems due to various reasons [11,12]. The vulnerability of nurses to abuse is likely to have a negative impact on their mental health, potentially affecting the care they provide [13]. In hospital settings, patients often express their frustrations toward nurses, which can be exacerbated in psychiatric settings due to unique treatment-related aspects [14].

Based on the above, the research problem might be elucidated by the lack of studies exploring the psychological consequences of violence from the perspective of psychiatric nurses. Therefore, the current study aims to explore the psychological consequences of violence as perceived by Saudi psychiatric nurses at Erada and the mental health complex in Jeddah City, Saudi Arabia.

## Materials and methods

Study design

This study used a descriptive, cross-sectional design to assess the psychological consequences of violence on psychiatric nurses [15]. A cross-sectional research design in medical research is a kind of observational research design that includes examining data from a demographic at a specific moment. Unlike other types of observational research, cross-sectional studies do not monitor individuals over time [15].

Sampling design and sample size

Sampling refers to the systematic process of selecting a representative subset, or sample, from a larger population to gather insights and estimate the characteristics of the entire population [16]. By examining and studying this subset, researchers can gain knowledge and make inferences about the broader population [16]. A convenience sampling technique was used for sample selection. The data collection questionnaire was distributed by WhatsApp or email as an electronic link to all nurses from the respective mental health facilities. This sampling technique allows each individual and every member of the population to have an equal chance or probability of selection. The population sample was taken from two hospitals with psychiatric departments in Jeddah, Saudi Arabia. A total of 300 psychiatric nurses, constituting all nurses working in the two psychiatric and mental health departments at the selected hospitals, are the accessible population. Based on this, the minimal sample size is estimated to be 107 psychiatric nurses, depending on the power analysis equation. Nurses with less than a month of experience in a psychiatry department would not be included in the study. In this study, a sample of 198 psychiatric nurses was recruited.

Inclusion and exclusion criteria

This study seeks to recruit registered nurses with at least three months of experience in a psychiatric or mental health department at Erada and Mental Health Complex in Jeddah, Saudi Arabia. Participants must be actively working in a psychiatric or mental health department at the facility and must express their willingness to participate in the study. Exclusion criteria include not being currently employed at Erada and Mental Health Complex in Jeddah, Saudi Arabia, not being actively engaged in psychiatric or mental health departments at the facility, having less than one month of experience in a psychiatry department, not being a registered nurse, and being unwilling to take part in the study.

Settings

The study was conducted in Erada and Mental Health Complex in Jeddah, Saudi Arabia, a 210-bed psychiatric facility with eight in-patient units, an administration building, a mosque, an outpatient and ER department, an auditorium, a gymnasium, a cafeteria, a library, recreational and occupational therapy facilities, maintenance shops, and a laundry, in July 2023. The complex also provides a long-term treatment program. Nursing is performed in different areas, including detoxification, rehabilitation, outpatient, extended care, female, and pre-admission units. Nurses evaluate and interpret the nursing process and standard of patient care. The range of services includes patient population, complexity of care, staffing and qualification of staff, and standards of practice. The nursing department determines the scope of care and its key functions and responsibilities.

Data collection procedure

The first step in the data collection procedure for the study was to obtain approval from the relevant authorities. The Institutional Review Board of Erada and Mental Health Complex approved the study (approval number: A01732). These authorities encompassed both the academic boards of the institutions where the research was being conducted and the administration of the hospitals that were the subject of the investigation. These hospitals included Erada and Mental Health Complex in Jeddah, Saudi Arabia [17]. Gaining these approvals was of utmost importance to ensure the study was conducted within ethical guidelines and that the rights and privacy of both the institutions and the participants were respected [17].

Once approval had been secured, the researchers embarked on a preliminary visit to the study site. This visit served dual purposes. Firstly, it allowed the researchers to familiarize themselves with the working environment and dynamics of the respective psychiatric departments [17]. This preliminary insight was invaluable for the subsequent stages of the study. Secondly, the visit provided the researchers with the opportunity to introduce the study in person to the potential participants [17]. Face-to-face communication greatly aided in accurately conveying the objectives and requirements of the study.

The preliminary site visit also facilitated the process of sample selection. During this visit, the researchers were able to ascertain the sample size based on the predefined inclusion and exclusion criteria [18]. Ensuring that the sample accurately represented the broader population of psychiatric nurses working in the respective hospitals was a pivotal aspect of the study [18].

Upon selection, each participant was then thoroughly informed about the study's objective, the data collection procedure, their individual role in the study, and their rights as participants [18]. The participants were also made aware of their right to withdraw from the study at any point without facing any consequences [18].

Following the comprehensive briefing, the participants were asked to provide their informed consent. This was a crucial step as it verified that the participant was fully cognizant of what their participation entailed and had willingly agreed to be a part of the study [19].

After obtaining informed consent, the data collection process commenced. The primary tool for data collection was a questionnaire, which was distributed to the participating nurses via WhatsApp or email in the form of an electronic link [19]. The questionnaire was meticulously designed to assess the psychological impact of workplace violence on psychiatric nurses. Throughout this entire process, stringent measures were taken to maintain the confidentiality and privacy of the data collected [19].

Research instrument

The study utilized a comprehensive and structured self-administered questionnaire to capture a broad spectrum of data about the participants [20]. First and foremost, basic demographic data was collected. This included the age, gender, and educational qualification of the respondents [20]. Detailed information about their professional background was also captured, such as their specific work experience in terms of years and the areas they specialized in [20].

The questionnaire also delved into a more personal aspect of the respondents' lives. It aimed to gather data on factors that could potentially impact their professional experiences and responses to workplace violence [21]. This included questions about marital status and whether or not the respondent had a spouse [21]. It also inquired about the respondents' social network, assessing the presence or absence of social support. Understanding the degree of satisfaction with personal life and social support networks is important, as it could influence how workplace violence is experienced and coped with [21].

The questionnaire also directly addressed experiences of violence in the workplace [22]. The respondents were asked whether they had ever experienced violence in their professional setting and to specify the type of violence if any. This could include verbal abuse, physical assault, or other forms of aggression [22]. It was important to understand the frequency, nature, and origin of such incidents, as well as the consequences they had on the individual, both in the immediate aftermath and in the long term [22].

Additionally, the questionnaire sought to understand the psychological impact of such incidents on the respondents [23]. This was accomplished through a series of questions that gauged emotional responses, stress levels, anxiety levels, and general mental well-being post the violent incident [23].

Furthermore, to ensure the reliability and validity of the instrument, the questionnaire was subjected to a pilot test among a small group of psychiatric nurses who were not part of the actual study [24]. Feedback was taken from this pilot group to refine the questionnaire before it was distributed among the main study group [24]. The questionnaire was also reviewed by a group of experts in the field to ensure its content validity [24].

The Cronbach alpha coefficient was used to assess the reliability of the questionnaire. This coefficient measures the internal consistency of a test or questionnaire [25]. The Cronbach alpha value for this questionnaire was found to be 0.87, considered very good [25]. This indicated that the questions in the questionnaire consistently measured the same underlying construct, thus making it a reliable tool for data collection [25].

Data analysis procedure

The collected data was analyzed using SPSS Statistics version 22 (IBM Corp. Released 2013. IBM SPSS Statistics for Windows, Version 22.0. Armonk, NY: IBM Corp.). The first step was data cleaning, which involved removing any incomplete or inconsistent responses from the dataset [26]. Descriptive statistics, including frequency, percentage, mean, and standard deviation, were then computed for the demographic data [26]. For the inferential statistics, t-tests and one-way ANOVA tests were conducted to identify significant differences between groups [27]. Correlation analyses were also conducted to determine the relationships between different variables, specifically to understand how different demographic and personal factors influenced the psychological impact of workplace violence on psychiatric nurses [27]. The significance level was set at p<0.05 for all tests.

## Results

Socio-demographic and baseline characteristics of the enrolled nurses

A total of 198 psychiatric nurses participated in this study. The majority of the participants were male (59.6%), with an average age of 37.7 years (SD=6.6). Educational qualification indicated that 68.2% of the participants had a diploma, 28.3% had a baccalaureate degree, and only 3.5% held a master's degree. In terms of marital status, the majority of the participants were married (76.8%), followed by single (21.2%) and separated (2%). Work experience showed that 54.5% of the nurses had more than 10 years of experience, 37.4% had 6-10 years of experience, and 8.1% had one to five years of experience. Regarding workplace violence, 80.8% of the participants reported experiencing violence in their workplace. Furthermore, 80.8% of the nurses reported the presence of social support for nurses facing workplace violence. Among those who reported social support, 15.2% were very satisfied, 37.9% were satisfied, 36.4% were neutral, 5.6% were dissatisfied, and 5.1% were very dissatisfied with the support provided (Table 1).

**Table 1:** Frequencies and percentages for the socio-demographic characteristics of the participants (N=198) BSN: Bachelor of Science in Nursing

Variable	F (%)
Gender	
Male	118 (59.6)
Female	80 (40.4)
Age (M±SD)	37.7±6.6
Educational qualification	
Diploma	135 (68.2)
Baccalaureate (BSN)	56 (28.3)
Master's degree	7 (3.5)
Marital Status	
Single	42 (21.2)
Married	152 (76.8
Separated	4 (2)
Work experience	
Less than one year	0 (0)
1-5 years	16 (8.1)
6-10 years	74 (37.4)
More than 10 years	108 (54.5)
Have you ever experienced violence in the workplace?	
Yes	160 (80.8)
No	38 (19.2)
Is there a presence of social support for nurses facing workplace violence?	
Yes	160 (80.8)
No	38 (19.2)
If yes, what is your degree of satisfaction with the support provided	
Very satisfied	30 (15.2)
Satisfied	75 (37.9)
Neutral	72 (36.40
Dissatisfied	11 (5.6)
Very dissatisfied	10 (5.1)

Factors related to violence in psychiatric settings

In this study, participants rated factors related to violence in psychiatric settings across two domains: patient-related factors and nursing-related factors. The patient-related factors domain included three items, with drug abuse having the highest mean score of 4.07 (SD=0.89) and ranked as the most significant factor related to violence in psychiatric settings. The type of psychiatric diagnosis had a mean score of 3.74 (SD=0.93) and was ranked second, while patients' previous violent behaviors had a mean score of 3.69 (SD=0.91) and were ranked fourth in terms of importance. The nursing-related factors domain had five items, with a lower level of experience in psychiatric care having the lowest mean score of 3.43 (SD=1.11) and ranked as the least important factor related to violence. This is less opportunity to experience violence relative to nurses with longer working careers. Lack of training in aggression control techniques had a mean score of 3.65 (SD=0.93) and was ranked sixth, followed by low tolerance levels of staff with a mean score of 3.62 (SD=0.92) and ranked seventh. Ineffective communication between nurses and patients had a mean score of 3.73 (SD=0.80) and was ranked third, while long-term shortage of psychiatric nurses had a mean score of 3.67 (SD=1.07) and was ranked fifth in terms of importance (Table 2).

**Table 2:** Means, standard deviations, and ranks of the factors related to violence in psychiatric settings (N=198)

Domain	Item	Mean	SD	Rank
Patient-related factors	Type of psychiatric diagnosis	3.74	0.93	2
	Patients' previous violent behaviors	3.69	0.91	4
	Drug abuse	4.07	0.89	1
Nursing-related factors	Lower level of experience in psychiatric care	3.43	1.11	8
	Lack of training in aggression control techniques	3.65	0.93	6
	Low tolerance levels of staff	3.62	0.92	7
	Ineffective communication between nurses and patients	3.73	0.80	3
	Long-term shortage of psychiatric nurses	3.67	1.07	5

Consequences of violence at the workplace as perceived by the psychiatric nurses

In this study, participants rated their distress related to difficulties experienced after stressful life events during the past seven days with respect to exposure to both physical and psychological violence by patients in healthcare systems. The distress was measured on a scale ranging from 0 (not at all) to 4 (extremely). The mean scores, standard deviations, and ranks for each item are presented in Table 3.

**Table 3:** Means, standard deviations, and ranks of the psychiatric nurses' responses to the Impact of Events Scale - Revised (IES-R) (N=198)

#	Item	M	SD	Rank
1	“Any reminder brought back feelings about it”	1.75	0.88	2
2	“I had trouble staying asleep”	1.40	1.00	17
3	“Other things kept making me think about it”	1.44	1.04	14
4	“I felt irritable and angry”	1.37	1.03	18
5	“I avoided letting myself get upset when I thought about it or was reminded of it”	1.57	1.09	7
6	“I thought about it when I didn’t mean to”	1.46	0.94	13
7	“I felt as if it hadn’t happened or wasn’t real”	1.51	0.98	10
8	“I stayed away from reminders about it”	1.68	1.10	3
9	“Pictures about it popped into my mind”	1.43	1.05	16
10	“I was jumpy and easily startled”	1.54	1.06	8
11	“I tried not to think about it”	1.63	1.03	6
12	“I was aware that I still had a lot of feelings about it, but I didn’t deal with them”	1.51	0.95	9
13	“My feelings about it were kind of numb”	1.64	1.05	4
14	“I found myself acting or feeling as though I was back at that time”	1.33	1.08	19
15	“I had trouble falling asleep”	1.29	1.06	22
16	“I had waves of strong feelings about it”	1.48	1.10	11
17	“I tried to remove it from my memory”	1.64	1.17	5
18	“I had trouble concentrating”	1.44	1.10	15
19	“Reminders of it caused me to have physical reactions, such as sweating, trouble breathing, nausea, or a pounding heart”	1.47	1.28	12
20	“I had dreams about it”	1.29	1.29	21
21	“I felt watchful or on guard”	1.33	1.20	20
22	“I tried not to talk about it”	2.10	1.18	1

The Impact of Event Scale - Revised (IES-R) was used to measure the psychiatric nurses' responses to a traumatic event. The mean scores and standard deviations were calculated for each item, and the items were ranked based on their mean scores. The highest mean score was found for item 22, "I tried not to talk about it," with a mean score of 2.10 (SD=1.18), indicating that nurses tend to avoid discussing the traumatic event. Item 1, "Any reminder brought back feelings about it," had the second-highest mean score of 1.75 (SD=0.88), indicating that reminders of the event trigger emotional responses. Item 8, "I stayed away from reminders about it," had a mean score of 1.68 (SD=1.10) and ranked third, indicating that nurses may try to avoid reminders of the traumatic event. The mean scores for items 7, 10, 13, and 17 were between 1.51 and 1.64, indicating that nurses may experience denial, emotional numbing, and attempts to remove the event from their memory. The mean scores for items 2, 3, 4, 6, 9, 11, 12, 14, 15, 16, 18, 19, 20, and 21 ranged from 1.29 to 1.48, indicating that nurses may experience symptoms such as insomnia, irritability, intrusive thoughts, concentration difficulties, physical reactions, and hypervigilance.

The means and standard deviations for the avoidance, intrusion, and hyperarousal subscales and the total score of the IES-R were calculated for 198 psychiatric nurses. The mean score for the avoidance subscale was 1.55 (SD=0.78), the highest mean score among the subscales and ranked first in terms of severity. The mean score for the intrusion subscale was 1.45 (SD=0.78), which was the second-highest mean score, and the mean score for the hyperarousal subscale was 1.41 (SD=0.81), which was the third-highest mean score. The total mean score for the IES-R was 1.40 (SD=0.72). The total score represents the overall severity of the psychiatric nurses' response to the traumatic event, and it is not ranked against the subscales (Table 4).

**Table 4:** Means, standard deviations, and ranks for avoidance, intrusion, and hyperarousal subscales and total score (N=198)

Subscale	Mean	SD	Rank
Avoidance	1.55	0.78	1
Intrusion	1.45	0.78	2
Hyperarousal	1.41	0.81	3
Total	1.40	0.72	-

## Discussion

What are the forms of psychological violence that psychiatric nurses are exposed to?

Workplace Violence

About 80.8% of psychiatric nurses reported experiencing violence in their workplace. This finding indicates that workplace violence is a significant problem for psychiatric nurses, consistent with previous research. A study by Basfr et al. [21] found that the prevalence of workplace violence against nurses in psychiatric hospitals in Saudi Arabia was 90.3%. The high prevalence of workplace violence highlights the need for effective interventions to prevent and manage violence in psychiatric settings.

Educational Qualification

About 68.2% of the participants had a diploma, 28.3% had a baccalaureate degree, and only 3.5% held a master's degree. This finding suggests that the majority of psychiatric nurses in the study had a relatively low level of educational attainment. This is consistent with previous research that has found that many psychiatric nurses have less education than nurses in other specialties [22]. The finding highlights the need for interventions that can improve the education and training of psychiatric nurses.

Social Support

About 80.8% of nurses reported the presence of social support for nurses facing workplace violence, and the majority of them were satisfied with the support provided. This finding suggests that social support is available to psychiatric nurses who experience workplace violence, which is important for their well-being and job satisfaction. The high level of satisfaction with the support provided suggests that the support is effective. This finding is consistent with previous research that has found that social support can buffer the negative effects of workplace violence on nurses' well-being [23].

Work Experience

About 54.5% of nurses had more than 10 years of experience, 37.4% had 6-10 years of experience, and 8.1% had one to five years of experience. This finding suggests that a significant proportion of psychiatric nurses in the study had extensive work experience, which is important for managing the challenges of working in psychiatric settings. However, the finding also suggests that a substantial proportion of nurses had relatively little experience, which may be a concern given the high prevalence of workplace violence. Previous research has found that nurses with more experience may be better equipped to manage workplace violence [24].

What are the factors related to psychological violence in psychiatric nursing?

Psychiatric nurses in the study rated factors related to violence in psychiatric settings across two domains: patient-related factors and nursing-related factors. Among the patient-related factors, drug abuse had the highest mean score of 4.07 (SD=0.89) and was ranked as the most significant factor related to violence in psychiatric settings. The type of psychiatric diagnosis had a mean score of 3.74 (SD=0.93) and was ranked second, while patients' previous violent behaviors had a mean score of 3.69 (SD=0.91) and were ranked fourth in terms of importance. Among the nursing-related factors, a lower level of experience in psychiatric care had the lowest mean score of 3.43 (SD=1.11) and ranked as the least important factor related to violence.

Lack of training in aggression control techniques had a mean score of 3.65 (SD=0.93) and was ranked sixth, followed by low tolerance levels of staff with a mean score of 3.62 (SD=0.92) and ranked seventh. Ineffective communication between nurses and patients had a mean score of 3.73 (SD=0.80) and was ranked third, while long-term shortage of psychiatric nurses had a mean score of 3.67 (SD=1.07) and was ranked fifth in terms of importance. The study found that drug abuse by patients was the most significant factor related to violence in psychiatric settings. This is consistent with previous research that has shown a strong association between substance abuse and violent behavior in psychiatric patients. The study also found that the type of psychiatric diagnosis and patients' previous violent behaviors were significant factors related to violence, which is in line with previous research [25,26].

Among nurses who had experienced violence, those with less experience in psychiatric care did not rate this as a significant factor related to violence. This finding is contrary to previous research that has suggested that less-experienced nurses are at higher risk of workplace violence due to a lack of confidence in handling challenging situations. However, it is important to note that less-experienced nurses may have had fewer opportunities to experience violence, which could confound this finding. The finding that ineffective communication between nurses and patients was a significant factor related to violence is consistent with previous research [27-29]. Poor communication can lead to misunderstandings and frustration, escalating into violent behavior. The study also found that a long-term shortage of psychiatric nurses was a significant factor related to violence, which is consistent with previous research that has shown that staffing levels and workload can contribute to workplace violence in healthcare settings.

What are the consequences of psychological workplace violence on psychiatric nurses?

Regarding the psychological consequences of violence as reported by the enrolled psychiatric nurses, the findings of the study showed that psychiatric nurses tend to avoid discussing the traumatic event they experienced, as indicated by the highest mean score of 2.10 (SD=1.18) for item 22, "I tried not to talk about it." Reminders of the traumatic event trigger emotional responses in nurses, as indicated by the second-highest mean score of 1.75 (SD=0.88) for item 1, "Any reminder brought back feelings about it." Nurses may try to avoid reminders of the traumatic event, as indicated by a mean score of 1.68 (SD=1.10) and ranking third for item 8, "I stayed away from reminders about it." Nurses may experience denial, emotional numbing, and attempts to remove the event from their memory, as indicated by mean scores between 1.51 and 1.64 for items 7, 10, 13, and 17. Nurses may try to avoid reminders of the traumatic event, as indicated by a mean score of 1.68 (SD=1.10) and ranking third for item 8, "I stayed away from reminders about it." In addition, nurses may experience denial, emotional numbing, and attempts to remove the event from their memory, as indicated by mean scores between 1.51 and 1.64 for items 7, 10, 13, and 17. The findings of this study suggest that psychiatric nurses who experience traumatic events tend to avoid discussing them and may try to avoid reminders of the event. These findings are consistent with previous studies that have found that avoidance is a common coping mechanism for individuals who have experienced traumatic events [29-30].

The high mean score for item 1, "Any reminder brought back feelings about it," indicates that reminders of the event may trigger emotional responses in nurses, consistent with previous research that has found that reminders of traumatic events can lead to increased emotional distress [18]. The finding that nurses may experience denial, emotional numbing, and attempts to remove the event from their memory is also consistent with previous research on trauma-related symptoms (e.g., American Psychiatric Association, 2013). The mean scores for items related to symptoms such as insomnia, irritability, intrusive thoughts, concentration difficulties, physical reactions, and hypervigilance are also consistent with previous studies that have found that individuals who have experienced traumatic events may experience a range of physical and psychological symptoms [21].

The findings of the present study showed that psychiatric nurses in this study experienced high levels of avoidance, intrusion, and hyperarousal in response to a traumatic event, with avoidance being the most severe. The mean scores for the subscales and total score of the IES-R indicate that the nurses experienced significant symptoms of post-traumatic stress disorder (PTSD). This finding is consistent with previous literature, which suggests that healthcare workers, including psychiatric nurses, are at high risk for exposure to traumatic events and subsequent development of PTSD symptoms. Previous studies have reported that healthcare workers often experience high levels of avoidance, intrusion, and hyperarousal in response to traumatic events. For example, a study [19] found that healthcare workers exposed to violence experienced high levels of PTSD symptoms, including avoidance, intrusion, and hyperarousal. Moreover, the finding that avoidance was the most severe symptom is also consistent with previous research. A study [15] found that avoidance was the most common symptom of PTSD among healthcare workers who experienced a traumatic event. Another study by Copeland and Henry [11] found that avoidance was the most severe symptom of PTSD among emergency department personnel who experienced workplace violence.

Limitations of the study

The study has several limitations that must be acknowledged. First, the research focused solely on psychiatric nurses at Erada and Mental Health Complex in Jeddah, Saudi Arabia, limiting the generalizability of the findings to a broader population of psychiatric nurses in different regions or healthcare settings. Second, the use of convenience sampling may introduce selection bias, potentially affecting the representativeness of the sample and the validity of the study's conclusions. Third, the cross-sectional design offers only a snapshot of the psychological consequences of workplace violence at one point in time, precluding an exploration of causal relationships or changes over time. Fourth, the reliance on self-reported data through questionnaires may be subject to response bias, social desirability bias, and a lack of objectivity. Fifth, the study's scope may not comprehensively capture all factors influencing psychiatric nurses' mental health, such as coping strategies, support systems, or organizational factors. Sixth, potential recall bias could impact the accuracy of data regarding past experiences of workplace violence. Seventh, ethical and reporting biases may have led to underreporting of sensitive or traumatic incidents. Finally, the specific cultural and healthcare context in Saudi Arabia may influence the psychological consequences of workplace violence, making the findings less directly applicable to other cultural settings. These limitations collectively underscore the need for cautious interpretation and consideration of contextual factors when applying the study's findings. Future research should aim to address these limitations and adopt diverse methodologies to deepen our understanding of this critical issue.

## Conclusions

The present study highlights several important findings regarding workplace violence, educational qualification, social support, work experience, factors related to violence, and response to traumatic events among psychiatric nurses. The high prevalence of workplace violence among psychiatric nurses emphasizes the need for effective interventions to prevent and manage violence in psychiatric settings. Moreover, the majority of psychiatric nurses in this study had a relatively low level of educational attainment, which suggests the need for interventions that can improve the education and training of psychiatric nurses. Additionally, social support was available to psychiatric nurses who experienced workplace violence, and the high level of satisfaction with the support provided suggests that the support is effective. The findings also revealed that drug abuse by patients was the most significant factor related to violence in psychiatric settings. In contrast, a lower level of experience in psychiatric care was rated as the least important factor related to violence, which is contrary to previous research. Psychiatric nurses who experienced traumatic events tended to avoid discussing them and may try to avoid reminders of the event. They also experienced high levels of avoidance, intrusion, and hyperarousal in response to a traumatic event, with avoidance being the most severe. These findings suggest the need for effective interventions to manage and prevent PTSD symptoms among psychiatric nurses.
